# The impact of radiotherapy in the treatment of desmoid tumours. An international survey of 110 patients. A study of the Rare Cancer Network

**DOI:** 10.1186/1748-717X-2-12

**Published:** 2007-03-07

**Authors:** Brigitta G Baumert, Martin O Spahr, Arthur Von Hochstetter, Sylvie Beauvois, Christine Landmann, Katrin Fridrich, Salvador Villà, Michael J Kirschner, Guy Storme, Peter Thum, Hans K Streuli, Norbert Lombriser, Robert Maurer, Gerhard Ries, Ernst-Arnold Bleher, Alfred Willi, Juerg Allemann, Ulrich Buehler, Hugo Blessing, Urs M Luetolf, J Bernard Davis, Burkhardt Seifert, Manfred Infanger

**Affiliations:** 1 Radiation Oncology, University Hospital Zurich, Switzerland; 2 Dept of Radiation Oncology (MAASTRO), GROW, University Hospital Maastricht, The Netherlands; 3 Dept. of Pathology, University Hospital Zurich, Switzerland; 4 Dept. de Radio-Oncologie, Centre des Tumeurs de l'Université Libre de Bruxelles, Belgium; 5 Radiation Oncology, University Hospital Basel, Switzerland; 6 Institute for Pathology, University Hospital Basel, Switzerland; 7 Radiation Oncology, Institut Català d'Oncologia, Barcelona, Spain; 8 Klinik und Poliklinik fuer Strahlentherapie, Erlangen, Germany; 9 Radiation Oncology, Oncologie Centre, Vrije Universiteit Brussels, Belgium; 10 Radiation Oncology, Ospedale S. Giovanni, Bellinzona, Switzerland; 11 Chirurgische Klinik, Kantonsspital Aarau, Switzerland; 12 Radio-Onkologie, Stadtspital Triemli, Zurich, Switzerland; 13 Dept. of Pathology, Stadtspital Triemli, Zurich, Switzerland; 14 Radiation Oncology, Kantonsspital St. Gallen, Switzerland; 15 Dept. of Radiation Oncology, University Hospital Bern, Switzerland; 16 Radiation Oncology, Kantonsspital Chur, Switzerland; 17 Dept. of Pathology, Kantonsspital Chur, Switzerland; 18 Chirurgische Klinik, Spital Schiers, Switzerland; 19 Chirurgische Klinik, Kantonsspital Glarus, Switzerland; 20 Dept. of Biostatistics, University Zurich, Switzerland; 21 Dept. of Hand-Plastic-and Reconstructive Surgery, University Hospital Zurich, Switzerland; 22 Praxis fuer Strahlentherapie, Solingen, Germany; 23 Dept. of Hand, Plastic and Reconstructive Surgery, Charite, University Medicine Berlin, Germany; 24 Pathology Clinics, Rikshospitalet-Radiumhospitalet Medical Center, Oslo, Norway; 25 Service de Radiothérapie, Clinique Saint Jean, Brussels, Belgium; 26 Deceased 1999

## Abstract

**Purpose:**

A multi-centre study to assess the value of combined surgical resection and radiotherapy for the treatment of desmoid tumours.

**Patients and methods:**

One hundred and ten patients from several European countries qualified for this study. Pathology slides of all patients were reviewed by an independent pathologist. Sixty-eight patients received post-operative radiotherapy and 42 surgery only. Median follow-up was 6 years (1 to 44). The progression-free survival time (PFS) and prognostic factors were analysed.

**Results:**

The combined treatment with radiotherapy showed a significantly longer progression-free survival than surgical resection alone (p smaller than 0.001). Extremities could be preserved in all patients treated with combined surgery and radiotherapy for tumours located in the limb, whereas amputation was necessary for 23% of patients treated with surgery alone. A comparison of PFS for tumour locations proved the abdominal wall to be a positive prognostic factor and a localization in the extremities to be a negative prognostic factor. Additional irradiation, a fraction size larger than or equal to 2 Gy and a total dose larger than 50 Gy to the tumour were found to be positive prognostic factors with a significantly lower risk for a recurrence in the univariate analysis. This analysis revealed radiotherapy at recurrence as a significantly worse prognostic factor compared with adjuvant radiotherapy. The addition of radiotherapy to the treatment concept was a positive prognostic factor in the multivariate analysis.

**Conclusion:**

Postoperative radiotherapy significantly improved the PFS compared to surgery alone. Therefore it should always be considered after a non-radical tumour resection and should be given preferably in an adjuvant setting. It is effective in limb preservation and for preserving the function of joints in situations where surgery alone would result in deficits, which is especially important in young patients.

## Background

Desmoid tumours are uncommon benign soft tissue neoplasms. Their incidence is reported to be 2–4/1.000.000 inhabitants in Finland [1,2] or 3% of all soft tissue tumours [3]. Aggressive fibromatoses or desmoid tumours are fibroblastic lesions with aggressive, infiltrative and destructive growth, which frequently recur if not widely resected [4]. Depending on the three major anatomic locations in which they arise, they are classified as: extra-abdominal fibromatosis, abdominal desmoid, occurring typically in women during or following pregnancy; and intra-abdominal fibromatosis, either a pelvic or mesenteric location. While most cases are sporadic, some are associated with familial adenomatous polyposis (FAP, Gardner's Syndrome) and these are most often intra-abdominal [5]. There are also cases of familial desmoid tumours at multiple sites, often involving one extremity, in patients without FAP. In both FAP and familial non-FAP tumours, mutations of the adenomatous polyposis coli (APC) gene on the long arm of chromosome 5 have been incriminated. The resultant loss of ability to degrade beta-catenin and elevated beta-catenin levels promotes fibroblastic proliferation [6]. In all settings and locations these fibroblastic proliferations are similar: variably cellular, often hypocellular ill-defined fascicles of fibroblasts and myofibroblasts lacking nuclear pleomorphism and showing little mitotic activity [7].

As fibromatoses do not metastasise, surgical radicality is often compromised when weighed against function preservation. It is the ill-defined margins of infiltration along septal planes that lead to recurrences. This necessitates mutilating operations, which may be avoided by adding radiotherapy to the treatment regimen. Relapse rates at 5 years after radiotherapy are reported as 33% [8,9]. Recent literature shows growing evidence that the addition of radiotherapy results in better local control than surgery alone independent of resection margin status [10,11]. This might support the hypothesis that with a combined treatment only modest surgical interventions may be needed, thus avoiding disfigurement. Additionally, radiotherapy alone may serve as a primary therapy and result in minor or no deficits for those patients whose tumours are un-resectable. Data for this study were obtained from European centres which are members of the "Rare Cancer Network" [12]. This study aims to contribute to an assessment of the therapeutic value of radiotherapy in the multimodal treatment of desmoid tumours.

## Patients and methods

### Patient selection

Departments of Surgery and Radiation Oncology of 14 centres within the Rare Cancer Network from 4 European countries participated in this study (Table 1). Departments of Pathology provided databases, but treatment data were collected from Surgery and Radiation Oncology only. In large Swiss centres, patients were discussed and treatment decisions taken centrally, for some patients part of treatment was done in smaller centres. A questionnaire concerning prognostic factors, postulated aetiology, treatment parameters, outcome, side-effects and follow-up was sent to the participating centres. The records of 140 patients were reported. All cases were histologically reviewed by an independent reference pathologist of the Pathology Department, University Hospital Zurich. After a histological review, only 110 of 140 patients were found to be eligible. They were treated between 1956 and 1997 and followed until 2001. Thirty patients were excluded because of a different diagnosis (e.g. malignant fibromyosarcoma, Dupuytren, juvenile fibromatosis) or insufficient information available for pathologic review. Data were reported and analyzed throughout the whole study anonymously using a coding system based on consecutive numbers per patient and per centre.

**Table 1:** Participating institutes

Dept*. of Radiation Oncology	**Country**	**No. patients**
Institute J. Bordet Brussels	Belgium	22
University Hospital Basel	Switzerland	21
University Hospital Zurich	Switzerland	15
Catalan Institute of Oncology Barcelona	Spain	15
Ospedale San Giovanni Bellinzona	Switzerland	4
University Hospital VUB Brussels	Belgium	4
University Hospital Erlangen	Germany	5
Stadtspital Triemli Zurich	Switzerland	3
Kantonsspital St. Gallen	Switzerland	3
University Hospital Berne	Switzerland	2
Kantonsspital Chur	Switzerland	1
		
**Dept. of Surgery**		
Kantonsspital Chur, Glarus and Schiers	Switzerland	7
Kantonsspital Aarau	Switzerland	5
Stadtspital Zurich	Switzerland	3

Abbreviation: * Dept.: departments

### General

The median follow-up period was 6 years (range 1 – 44 years), for 4 patients data were insufficient for follow-up. Thus, 106 patients were evaluable for the survival analysis and 110 patients for descriptive statistics. One patient had a bifocal disease (left and right arm), where one tumour was treated with surgery alone and the other with surgery and post-operative radiotherapy. Seventy-eight patients were female, 32 male. Fifty-nine percent of all recurrences appeared during the first 2 years and 82% within 5 years of treatment. One patient died of intercurrent disease.

Sixty-eight patients were treated with surgery and post-operative radiotherapy (Sx+RT), 42 with surgical resection alone (Sx). An overview of the patients' characteristics and distribution between both groups is given in Table 2. The two treatment groups were statistically comparable in terms of age, gender, resection margins and aetiological factors. The resection margin status at first operation shows a significant difference in frequencies, mainly for R1 resection, however, numbers are small. For further statistical evaluation, margins of R0 and R1 are grouped together as radical resection, and R2, R3 and unknown as non-radical resection (Radical: 59% for the Sx+RT group, 55% for Sx, non-radical: 41% for Sx+RT, 44% for Sx). The number of re-operations was higher in the group which received postoperative radiotherapy (p < 0.0001). The frequencies of tumour localization were differently distributed within both groups, mainly for tumour localization of abdominal wall and extremities. Tumours of the trunk include the following localizations: 4 tumours in the breast, 5 intra-thoracic, 12 intra-peritoneal and 1 retro-peritoneal tumour. The median age was 33 years (range 1– 78). The majority of patients were between 21 – 40 years old at the time of the first treatment. Patients were regularly followed-up, clinically and by imaging according to each department's scheme. Imaging was by MRI scans since its general availability.

**Table 2:** Patients' characteristics

**Factor**	**Total No. **	**Surgery and radiotherapy**	**Surgery alone**	**P-value**
Follow-up (yrs)	0 (1–44)	7.8 (0.6 – 44.3)	3.1 (0.1 – 24.9)	< 0.0001
Age (yrs)	33 (1–78)	33 (1–78)	32 (1–65)	n.s.*
Gender				
Male	32 (29%)	20 (29%)	12 (29%)	n.s.
Female	78 (71%)	48 (71%)	30 (71%)	
Tumour localization				0.003
Abdominal wall, testicular sheath	25 (23%)	10 (15%)	15 (36%)	
Head and neck	7 (6%)	6 (9%)	1 (2%)	
Extremities, shoulder girdle, hip	56 (51%)	43 (63%)	13 (31%)	
Trunk, pelvis, breast	22 (20%)	9 (13%)	13 (31%)	
Resection margin				0.004
R0	16 (14%)	4 (6%)	12 (29%)	
R1	47 (43%)	36 (53%)	11 (26%)	
R2	20 (18%)	14 (21%)	6 (14%)	
R3	13 (12%)	7 (10%)	6 (14%)	
Unknown	14 (13%)	7 (10%)	7 (17%)	
Number re-operations	2.3/2.0 (1–12)	2.65/2.0 (1–7)	1.74/1.0 (1–12)	< 0.0001
Radiotherapy dose	56.5/59.4	57.4/59.4	-	-
> 50 Gy	58 (53%)	58 (85%)	-	
< = 50 Gy	8 (8%)	8 (12%)	-	
No dose, unknown	44 (39%)	2 (3%)	-	
Fraction size	2.28/2.0	2.29/2.00	-	-
>= 2 Gy	40 (36%)	40 (59%)	-	
< 2 Gy	15 (14%)	15 (22%)	-	
No dose, unknown	55 (50%)	13 (19%)	-	
Indication radiotherapy				
Adjuvant	24 (22%)	24 (35%)	-	-
At recurrence	39 (35%)	39 (57%)	-	
Primary	5 (4%)	5 (7%)	-	
Etiological factor				
Yes	44 (40%)	29 (43%)	15 (36%)	n.s.
No	66 (60%)	39 (57%)	27 (64%)	

Numbers present: median (range), mean/median (range), no. (%).

*Abbreviation*: *n.s.: not significant

### Treatment

All patients had surgery and none had radiotherapy alone. Baseline operations were reported between 1956 and 1996. Radiation was given between 1964 and 1997. Five patients had a wide biopsy and were classified as having macroscopic residual margin. Surgical margins were defined as follows: wide excision with a margin > 1 cm (R0), margin < 1 cm (R1), microscopic residual tumour (R2) and macroscopic residual tumour (R3) (Table 3). The indications for radiotherapy were primary radiotherapy after biopsy or wide biopsy only, postoperative adjuvant radiotherapy or in case of recurrence. Radiotherapy techniques and dose specifications differed over the time span of this study and between participating centres. In order to have comparable therapeutic nominal doses, all doses were recalculated and reported according to ICRU recommendations [13]. Two cases could not be considered for dose recalculation, because specifications about radiotherapy doses were not available. In 62 cases the radiotherapy technique used was external beam radiotherapy with photons and/or electrons from a linear accelerator or Cobalt unit. In 4 cases brachytherapy was used as a boost and in 3 cases orthovoltage X-rays were used. For two patients a split course technique was used. Fraction size ranged from 1.5 Gy to 3 Gy (median 2.28 Gy). The median total radiation dose was 59.4 Gy in 29 fractions (Range 3.4 Gy–73.7 Gy). Eight patients received 50 Gy or less. For details see Table 2.

**Table 3:** Recurrence rates after first operation according to resection margin status

Resection margin	Recurrence rate No. patients (%)	Surgery and radiotherapy No. (%)	Surgery alone No. (%)
R0	5/16 (31)	2/4 (50)	3/12 (25)
R1	30/47 (64)	27/36 (75)	3/11 (27)
R2	11/20 (55)	9/14 (64)	2/6 (33)
R3	7/13 (54)	5/7 (71)	2/6 (33)
RX	7/14 (50)	5/7 (71)	2/7 (29)

R0, wide excision; R1, margin < 1 cm; R2, microscopic residual tumour; R3, macroscopic residual tumour; RX, unknown.

### Statistical analysis

Data were analysed using the SPSS (version 13 for Mac OS X, SPSS Inc. IL) and the Stata software packages (Release 8.2, Stata Corp.). Groups were compared using Fisher's Exact test and the Mann-Whitney test when appropriate. The progression-free survival (PFS) was calculated beginning with the date of first surgery until recurrence or last follow-up. The overall progression-free survival was analysed using Kaplan-Meier curves and the log-rank test. For this analysis there is one endpoint per patient, i.e. the outcome at the last follow-up after all therapeutic events independent of the order or indication of treatments (i.e. several operations before radiotherapy, or the number of resections before and after radiotherapy etc). Progression-free survival between multiple events (i.e. multiple recurrences and treatments in one patient) was analyzed using a Cox Regression Model with shared frailty. This way, the effect of different radiotherapy treatments during the course of the disease due to recurrences within the same patient can be analyzed taking into account the different aggressiveness of tumours and the correlation between recurrences within the same patient. In a Cox Regression Model with shared frailty (Stata, procedure stcox), frailties are gamma-distributed latent random effects that enter multiplicatively in the hazard. Those frailties are shared by (and thus are constant for) all events within the same patient. The variance of frailties is estimated by iterative maximum profile log-likelihood. Univariate and multivariate analyses of multiple events were performed using a Cox Regression Model with shared frailty to determine the possible prognostic factors of gender, age, aetiology, resection margin, tumour localization, radiation therapy, radiotherapy indications, total radiotherapy dose and fraction size. A result was significant if p < 0.05.

## Results

### Descriptive analysis – Surgery

As follow-up data for 4 patients were missing, 38 patients were available for analysis. The number and percentages of recurrences after the first operation are shown in Table 3. The recurrence rate after the first surgical resection was 32% (12/38). Recurrence rates are lower for patients treated with surgery alone after the first resection (selection bias in favour of the surgery alone group). The lowest recurrence rate (31%) is found after wide radical resection (R0). Fourteen out of 42 patients had between 1 and 12 re-operations (mean/median 1.74/1.0). For 56 patients with a tumour located in the extremities, 13 were treated with surgery alone. Amputation was necessary for 3 of them (23%). One patient was disease-free after amputation, the remaining two patients relapsed and were treated with radiotherapy and were progression-free thereafter.

### Descriptive analysis – Radiotherapy

Of the 68 patients in the RT group, 22 patients were irradiated after the first operation, 25 after the first recurrence and 21 after the second or further recurrences. Seventeen of the 68 patients (25%) had local failures after post-operative irradiation with a median dose of 55.6 Gy (Range: 3.4 – 68 Gy). Recurrences were seen at the field borders in 7 cases and within the field in 10 cases. In 11 of those 17 cases (65%), recurrences were seen in areas where the dose was less than 50 Gy. Of those 17 patients, 11 were re-operated after irradiation, the tumour recurring in 3 patients and persisting in one thereafter. Seven patients were re-irradiated with a median dose of 50 Gy (Range: 40 – 65 Gy), 3 of them recurred. In all 43 patients treated with post-operative radiotherapy for a tumour located in the limbs, the extremities could be preserved.

### Descriptive analysis – Tamoxifen

Ten patients received an additional therapy with Tamoxifen: one patient after surgery, 4 patients for recurrence after irradiation, and 5 patients in combination with radiotherapy. Only one patient responded to Tamoxifen therapy. In 3 cases the tumour progressed under Tamoxifen therapy.

### Descriptive analysis – Aetiology

Aetiological factors were reported for 44 cases. These were the site of a previous trauma or an operation in 18 cases, pregnancy in 17 cases and the Gardner-Syndrome in 9 cases. No significant difference in the PFS between patients with and without known aetiological factors was found (Table 4).

**Table 4:** Analysis of prognostic factors for progression-free survival (Cox proportional hazard with frailty)

**Factor**	**Univariate**			Multivariate*		
	HR	(95% CI)	p-value	HR	(95% CI)	p-value

Radiation therapy	0.19	(0.11–0.31)	< 0.001	0.21*	(0.13 – 0.34)	< 0.001
Radiotherapy dose (>50 Gy)	0.60	(0.38–0.97)	0.028			
Fraction size (≥ 2 Gy)	0.59	(0.37–0.95)	0.036			
Resection margins*	1.07	(0.72–1.58)	n.s.			
Indication radiotherapy						
Adjuvant radiotherapy	0.42	(0.25–0.72)	0.002			
Radiotherapy at recurrence	2.69	(1.63–4.41)	< 0.001			
Primary radiotherapy	0.36	(0.11–1.15)	n. s.			
Tumour localization						
Head-neck	0.96	(0.46–2.00)	n.s.			
Trunk	0.67	(0.37–1.18)	n.s.			
Abdominal wall	0.42	(0.21–0.85)	0.017	0.28*	(0.15 – 0.53)	< 0.001
Extremities*	2.5	(1.68–3.62)	< 0.001			
Potential etiological factors	0.90	(0.61–1.32)	n.s.			
Gender (male)	0.95	(0.62–1.45)	n.s.			
Age (years)	0.99	(0.98–1.00)	n.s.			

*Without frailty calculated because Cox proportional hazard did not converge.

*Abbreviations*: HR: hazard ratio; Cl: Confidence interval; n.s. : not significant.

### Descriptive analysis – Toxicity

Side effects of treatment were reported for 76 patients only. Toxicity reported after surgery was as follows: no side effects (8 patients), pain (4), malabsorption syndrome (4), stiffening of joints (3), paresis (2), paraesthesia (1), fistula (1), ileus (1), lymph oedema (1), phantom pain (1), skin erythema (1), scar herniation (1), screw migration (1). Late side effects after combined surgery and radiotherapy were: stiffening of joints (22), hyper-pigmentation (11), paraesthesia (9), pain (6), paresis (6), skin ulceration (2), colon irritabile (1), ileus (1), scoliosis (1), scar-herniation (1), xerostomy (1). No radiotherapy-induced sarcoma was reported.

### Survival analysis – Overall outcome

**Figure 1:**
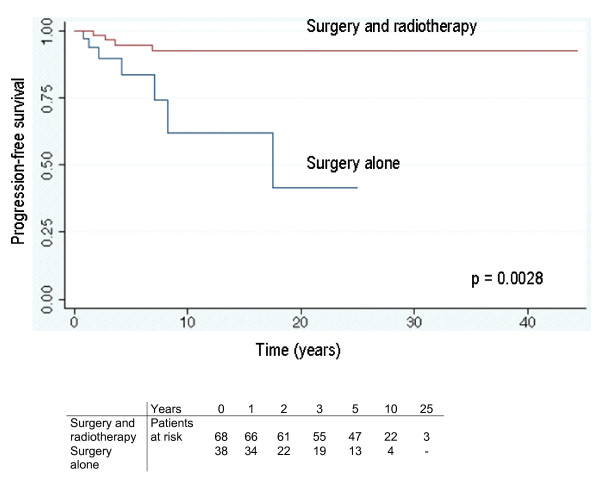
Overall progression-free survival at the last reported follow-up (Kaplan-Meier curves).

**Figure 2:**
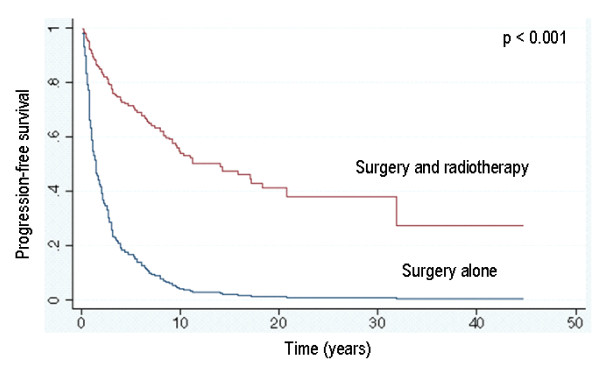
Progression-free survival taking multiple recurrent events per patient into account (Cox proportional hazard regression with shared frailty).

The overall outcome for the whole group after all therapeutic interventions (independently of number and order of treatments) showed progressive disease (7 patients), stable disease (9 patients) and no evidence of disease (94 patients). Figure 1 shows the overall outcome as endpoint with the tumour status as reported at the last follow-up. For patients treated with surgery and radiotherapy the PFS was 95 % and 93 % at 5 and 10 years respectively. For patients treated with surgery alone the PFS was 84% and 62% at 5 and 10 years, respectively. The difference was statistically significant (p = 0.0028). In order to answer the question of the role of radiotherapy in a combined treatment setting, an event-related analysis was performed. This analysis, which uses the Cox Regression Model with shared frailty (see statistics section above), looked at a presumed additional tumour-related risk factor per patient resulting in multiple recurrences after surgery alone or after combined treatment. This resulted in a different time-relationship between surgery and radiotherapy for each patient. The "shared frailty" model takes this into account. The progression-free survival was significantly better for patients who had received radiotherapy (p < 0.001) (Fig. 2).

### Survival analysis – Prognostic factors

The univariate analysis of possible prognostic factors revealed a significantly lower risk of recurrence related to the following factors: additional irradiation, a fraction size of ≥ 2 Gy with a hazard rate of 60%, a total dose > 50 Gy with a hazard rate of 59% (p = 0.028, Table 4). In multivariate analysis radiotherapy treatment and tumour localization in the abdominal wall were independent positive prognostic factors (Table 4). The comparison of adjuvant post-operative radiotherapy versus radiotherapy at recurrence found adjuvant radiotherapy to be significantly better (p < 0.001). Age was not a prognosticator. A more advanced age does not reduce the risk for a desmoid tumour. No age relation was found.

## Discussion

The optimal treatment for patients with aggressive fibromatosis remains unclear. Desmoid tumours are slowly proliferating tumours. The ultimate treatment goal is tumour control as the probability of dying from aggressive fibromatosis is relatively low. Patients with an intra-abdominal desmoid tumour are at a higher risk of dying of local tumour progression or of side-effects due to surgical or combined treatment. To achieve local control may be a challenge in these patients. Many have a local recurrence, often multiple recurrences, within the site of the primary tumour. Recurrence rates may differ with time and treatment modalities used. This makes it difficult to evaluate the value of adjuvant treatment, as radiotherapy could, for example, be given post-operatively or after a recurrence. The same patient could have had several resections and radiotherapy at some point in time. For this reason we have performed, in addition to the classic actuarial analysis (with the last follow-up as endpoint) a Cox Hazard Frailty Analysis. The classic analysis does not take into account a possible tumour and patient related risk, where some tumours keep recurring after the same primary treatment, whereas the Hazard Frailty Analysis takes into account the time-related probability of occurrence of failure and considers as such each patient individually.

### Surgery

Wide surgical excision is considered to be the standard treatment and can result in a cure. Cure is defined as no tumour progression or relapse. Published data indicate that the likelihood of local recurrence after surgery alone is high with reported recurrence rates ranging from 20% to 90% [3,14-20]. The local recurrence rate of 32% observed in this study was therefore low. Recurrence rates of up to 68% after resection have been described if positive resection margins are present [9,16,19]; whereas a local control of 85% in the case of a R0 excision can be reached [21]. In contrast, to what has been observed in this study, Reitamo et al found a lower recurrence rate after incomplete resection (17%) compared to a wide excision (24%) [2]. In an analysis of surgical margins between wide and microscopic complete resection we found only small differences. The reasons for these conflicting results are presumably due to: a selection bias in favour of the surgery alone group (in the survival analysis the recurrence rate is significantly lower for irradiated patients); the retrospective nature of the evaluation (based mainly on the surgeon's description of radicality) and the fact that mainly Radiation Oncology departments participated in this study. As a result, many patients with no recurrence after radical resection were not included in the study.

### Radiotherapy

Radiotherapy is a viable treatment option for desmoid tumours. This was shown as early as 1928 by J. Ewing [22]. Our data for local control after radiotherapy (75%) lie near the higher range of published data (69–80%) [9,10,18,21,23-27]. Local control is, independent of tumour status (primary or recurrent) and resection margin (negative versus positive), significantly increased if radiotherapy is added [11,28]. Post-operative adjuvant radiotherapy was significantly better than radiotherapy at recurrence. Recurrences reported after radiotherapy occurred within the field in 54% of the cases, and at the field border in 30%, out of the field in 16% and in areas irradiated with doses less than 50 Gy in 72% of the cases [11,29,30]. In this analysis, 61% (11/18) of the recurrences after irradiation were seen at the field border or in areas receiving doses less than 50 Gy. We therefore support the recommendation of other investigators to add wide radiation field margins of at least 5 cm in the direction of possible infiltrative growth [10,27].

To date there is insufficient published evidence to support a dose related effect. Recurrences after radiotherapy have been reported with doses > 60 Gy [9,31]. Although some investigators [32] could not demonstrate an improved tumour control rate for doses exceeding 50 Gy, we and others have found a significantly better local control for doses > 50 Gy (p = 0.028) [8,33].

### Follow-up

Most recurrences occur within 5 years [8-10,23,29]. Other workers are of the opinion that an earlier endpoint for evaluation is acceptable as 80% of all recurrences appear within the first two years of treatment [17,19,20,33,34]. In this study, 59% of all recurrences appeared during the first 2 years and 82 % during the 5 years following treatment. We detected recurrences at up to 20 years. For this reason and to our knowledge, this study has the longest reported range of follow-up (44 years) we would suggest that a longer mean follow-up than 5 years is advisable.

### Toxicity

Side effects reported in this study were not complete. No difference between side effects after surgery and after radiotherapy could be demonstrated. For this reason, after a median follow-up of 6 years, no secondary malignant tumours after radiotherapy have been reported, which may be expected in such a population treated at a young age. However, this may still be observed after a longer mean follow-up.

### Pharmacologic agents

Several systemic therapies have been proposed for the treatment of desmoid tumours. Responses to anti-estrogens [35-37], to non-steroidal and steroidal anti-inflammatories, and to cytotoxic chemotherapeutics have been reported [38-41]. Only one out of 10 patients (10 %) in this study treated with Tamoxifen showed a tumour response.

### Prognostic factors

None of the prognostic factors such as gender, pregnancy or Gardner's syndrome described in the literature [2,7,24] could be confirmed. Furthermore, we found no prognostic influence of age, whereas tumour localization was found to be a significant prognostic factor. A significant difference between trunk and extremities has been reported, with localization in the trunk having a better prognosis [10,15,28]. Tumours of the abdominal wall compared with tumours of the extremities showed a significantly better prognosis both in the univariate and multivariate analysis. A possible reason for this result is the better resectability of tumours in the abdominal wall. Anatomic structures are the limiting factors in extremities. However, an analysis of the surgical margins in these two regions did not support this hypothesis (data not shown). Another explanation could be the uneven distribution of patients with a tumour in the abdominal wall in the two treatment groups: the percentage of patients treated by surgery alone being higher. This finding is partly due to the fact that wide excision is the recommended first treatment approach. Patients are often referred to Radiation Oncology centres for treatment only after they had experienced multiple recurrences. Our data reflect this by the significantly higher number of re-operations found in the radiotherapy group. Last but not least, tumours of the abdominal wall may represent a different biologic behaviour. A first hint of this has been reported for Familial Adenomatous Polyposis (FAP) related desmoid tumours. Abdominal desmoids comprised the majority of FAP desmoids and extra-abdominal desmoids comprised the majority of non-FAP desmoids (P < 0.001) [42]. FAP desmoids may be genetically different. Based on our data, however, we could show no final proof for the factors of resectability or aetiology as a reason for favourable outcome in patients with a desmoid tumour of the abdominal wall.

Additionally, adjuvant postoperative radiotherapy was a positive prognosticator for PFS if compared to radiotherapy at recurrence. The addition of radiotherapy at an earlier time point of the disease may be advisable. We looked at the fraction size under the hypothesis that as desmoid tumours are slow growing tumours originating from fibroblasts, and thus may need a higher single fraction size. The use of daily fractions ≥ 2 Gy reduced the hazard for a tumour recurrence to 60%. To our knowledge this finding concerning the fraction size for radiotherapy of aggressive fibromatosis has not been reported in the literature.

## Conclusion

Wide resection remains the primary therapy, but, as this study shows, in certain situations adjuvant post-operative radiotherapy is a must in the treatment of aggressive fibromatoses. Radiotherapy should be part of the treatment concept for patients with non-radical tumour resection after primary surgery or at first recurrence, as well as for limb preservation. Radiotherapy should be considered early in the treatment concept because adjuvant post-operative radiotherapy improves local tumour control. The total dose applied should be above 50 Gy. Our data might indicate that it could be beneficiary to use fraction sizes ≥ 2 Gy. The radicality and the number of re-operations may be modified: adjuvant radiotherapy seems to compensate for positive resection margins and could therefore reduce the recurrence rate and avoid mutilating operations in this predominantly young patient group. However, the risk of a radiation induced tumour should be considered when treating young patients. A "cost-risk" estimation, whether the cost of a loss of a limb or more (e.g. as in a hemi-pelvectomy) versus the risk of a malignant tumour should be taken into account for each individual treatment decision.

Although it would be difficult to realize because of the rarity of these tumours, the contribution of radiotherapy to the treatment of desmoid tumours can only be answered by a prospective randomised clinical trial in a defined patient group, especially as modern three-dimensional radiotherapy treatment planning and the use of functional imaging may give a better indication of the incidence of recurrences and side effects.

## Competing interests

The author(s) declare that they have no competing interests.

## Authors' contributions

BGB: Designed and conducted the study, conducted data evaluation, wrote the article

MOS: Collected data, updated the follow-up, built the database, wrote first outline of the manuscript

AvH: Reviewed the pathology of all patients = reference pathologist.

SB: Support with data collection, entry of patients, critical review of the manuscript.

CL: Support with data collection, entry of patients.

KF: Support with pathological review of a subgroup of patients.

SV: Data collection, entry of patients, critical review of the manuscript.

MJK: Data collection, entry of patients, critical review of the study design and questionnaires.

GS: Data collection, entry of patients, critical review of the manuscript.

PT: Data collection, entry of patients, critical review of the manuscript.

HKS: Data collection, entry of patients, critical review of the manuscript.

NL: Support with data collection, entry of patients.

RM: Support with data collection and pathology review.

GR: Data collection, entry of patients, critical review of the manuscript.

EAB: Data collection, entry of patients.

AW: Data collection, entry of patients

JA: Support with data collection.

UB: entry of patients.

HB: entry of patients.

UML: Participated in the design of the study, supported first statistical evaluation and data collection (building of the database).

JBD: Recalculated all radiotherapy doses into actual used doses according to ICRU. Critical review of the manuscript.

BS: Performed the statistical analysis, helped with drafting of the manuscript.

MI: Support with data collection for the surgical aspects of the study, critical review of the manuscript.

All authors read and approved the final manuscript
